# The Cortical Signature of Amyotrophic Lateral Sclerosis

**DOI:** 10.1371/journal.pone.0042816

**Published:** 2012-08-06

**Authors:** Federica Agosta, Paola Valsasina, Nilo Riva, Massimiliano Copetti, Maria Josè Messina, Alessandro Prelle, Giancarlo Comi, Massimo Filippi

**Affiliations:** 1 Neuroimaging Research Unit, Institute of Experimental Neurology, Division of Neuroscience, San Raffaele Scientific Institute, Vita-Salute San Raffaele University, Milan, Italy; 2 Department of Neurology, Institute of Experimental Neurology, Division of Neuroscience, San Raffaele Scientific Institute, Vita-Salute San Raffaele University, Milan, Italy; 3 Biostatistics Unit, IRCCS-Ospedale Casa Sollievo della Sofferenza, San Giovanni Rotondo, Italy; 4 Neurology Unit, Ospedale Maggiore, Crema, Italy; Baylor College of Medicine, Jiao Tong University School of Medicine, United States of America

## Abstract

The aim of this study was to explore the pattern of regional cortical thickness in patients with non-familial amyotrophic lateral sclerosis (ALS) and to investigate whether cortical thinning is associated with disease progression rate. Cortical thickness analysis was performed in 44 ALS patients and 26 healthy controls. Group differences in cortical thickness and the age-by-group effects were assessed using vertex-by-vertex and multivariate linear models. The discriminatory ability of MRI variables in distinguishing patients from controls was estimated using the Concordance Statistics (C-statistic) within logistic regression analyses. Correlations between cortical thickness measures and disease progression rate were tested using the Pearson coefficient. Relative to controls, ALS patients showed a bilateral cortical thinning of the primary motor, prefrontal and ventral frontal cortices, cingulate gyrus, insula, superior and inferior temporal and parietal regions, and medial and lateral occipital areas. There was a significant age-by-group effect in the sensorimotor cortices bilaterally, suggesting a stronger association between age and cortical thinning in ALS patients compared to controls. The mean cortical thickness of the sensorimotor cortices distinguished patients with ALS from controls (C-statistic ≥0.74). Cortical thinning of the left sensorimotor cortices was related to a faster clinical progression (r = −0.33, p = 0.03). Cortical thickness measurements allowed the detection and quantification of motor and extramotor involvement in patients with ALS. Cortical thinning of the precentral gyrus might offer a marker of upper motor neuron involvement and disease progression.

## Introduction

Amyotrophic lateral sclerosis (ALS) is the most common adult-onset motor neuron disease, which is characterized by degeneration of both upper (UMN) and lower motor neurons [1]. In ALS patients, gross brain pathology can exhibit cortical atrophy of the precentral gyrus and contiguous cortices [2], [3]. Histopathology shows loss of giant pyramidal cells of Betz and surrounding motor neurons of the motor cortex, cranial nerve nuclei, and spinal cord anterior horn cells [2]. Degeneration of the corticospinal tracts in the anterior and lateral columns of the spinal cord is also evident [2]. Affected motor neurons often contain characteristic inclusions in the perikarya, dendrites and axons [2].

Despite many neuropathological studies describing loss of giant pyramidal cells of Betz within cortical layer V of the primary motor cortex, a quantitative measurement of cortical neuronal loss in postmortem ALS brains is challenging and may be unrevealing [3]. In addition, ALS pathology studies have not examined systematically extra-motor cortical areas [4]. MRI is sensitive to cortical atrophy, and several studies have demonstrated a distributed pattern of brain grey matter (GM) loss in ALS patients [5]. Voxel-based morphometry (VBM), which allows to measure GM density as a proxy for cortical volume, has been shown to be sensitive to extra-motor cortical atrophy in ALS [5], but not to that of primary motor regions [6], [7], [8], [9], [10], [11]. Recently, the width of the cortical layer that covers the surface of the brain, referred to as cortical thickness [12], has been assessed in a variety of disorders [13], [14], [15]. A few studies have investigated cortical thickness in ALS patients showing a significant thinning of the primary motor cortex [16], [17], [18]. Cortical thinning in temporal regions was related to faster clinical progression in one study [18]. However, the pattern of extra-motor cortical thinning has not been fully defined yet. Furthermore, the cortical association of damage with previous disease progression is still relatively unknown.

The objective of this study was to investigate the pattern of cortical thickness topography in patients with non-familial ALS compared with healthy controls. To this aim, we first used an exploratory analysis across the entire cortex to determine whether ALS is associated with a cortical signature of thinning in specific motor and extra-motor regions. Then, using a region of interest (ROI) analysis, vulnerable cortical regions located in both the sensorimotor and the “cognitive” frontal and temporal brain areas were defined, and the diagnostic accuracy in distinguishing ALS patients from healthy individuals was investigated. Finally, we explored whether cortical thickness was associated with age and the rate of disease progression, which was defined as the ratio of present disability and disease course [19]. We hypothesized that patients with ALS would present a reduced cortical thickness in the motor, prefrontal and temporal areas of the cortex, and that the cortical thinning would be more severe in those patients who have experienced a more rapid disease progression.

## Results

### Whole brain cortical thickness differences

ALS patients showed a decreased whole brain average cortical thickness (mean 2.07 mm±0.26, range: 1.58–2.47 mm) compared to controls (mean 2.18 mm±0.17, range: 1.81–2.40) (p = 0.047).

### Regional cortical thickness differences: vertex-by-vertex analysis

**Figure 1:**
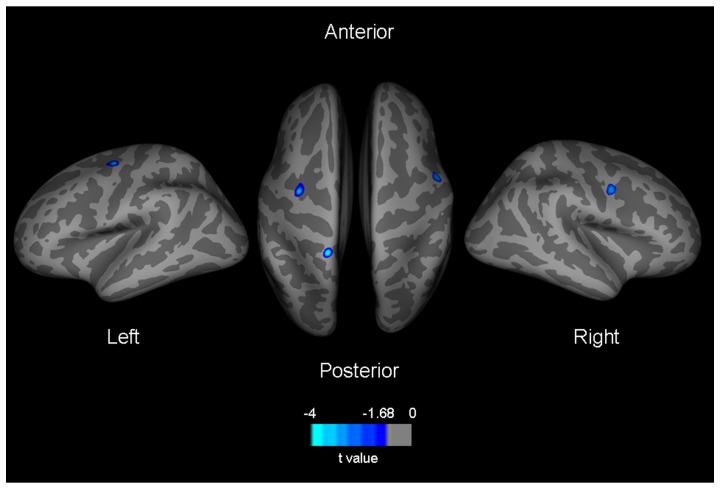
Vertex-by-vertex analysis: regions of significantly thinner cortex in patients with amyotrophic lateral sclerosis compared with healthy controls represented on an averaged brain map. Color bar represents t values (p<0.05, uncorrected for multiple comparisons).

Whole brain, vertex-by-vertex analysis showed that, compared with controls, ALS patients had a thinning of the left superior part of the precentral sulcus (x −31, y −10, z 49; p = 0.05), left paracentral gyrus (x −18, y −44, z 72; p = 0.01), and right precentral gyrus (x 56, y −1, z 39; p = 0.05) (p<0.05, uncorrected for multiple comparisons; Figure 1). The results did not change when the analysis was performed in limb-onset ALS patients compared with healthy controls (data not shown).

### Regional cortical thickness differences: ROI analysis

**Figure 2:**
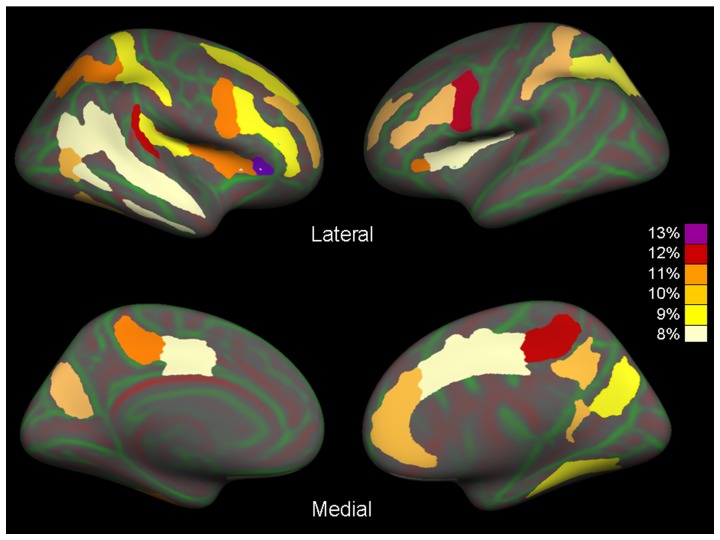
Cortical regions showing a significant cortical thinning in patients with amyotrophic lateral sclerosis compared with healthy controls overlaid on the lateral (top row) and medial (bottom row) views of the left and right hemispheres. Regions are color-coded according to the percent difference of cortical thickness between the two groups.

In the left hemisphere (Table 1; Figure 2), ALS patients showed a significant thinning (p<0.05, False-discovery rate [FDR]-corrected for multiple comparisons) of: a) frontal lobe (average thinning ranging from 8 to 12%), comprising the inferior part of the precentral sulcus, inferior and middle frontal sulci, and medial orbital sulcus; b) limbic lobe, comprising the left cingulate sulcus (marginal branch, thinning of 11%) and middle-posterior cingulate gyrus and sulcus (pMCC, average thinning = 8%); c) insula, comprising the circular (average thinning = 8%) and lateral (average thinning = 11%) sulci; d) temporal lobe, comprising the anterior transverse collateral sulcus (average thinning = 11%); and e) parietal and occipital areas (average thinning ranging from 8 to 10%), comprising the postcentral sulcus, intraparietal and transverse parietal sulci, and parieto-occipital sulcus. In the right hemisphere (Table 2; Figure 2), ALS patients showed a cortical thinning (p<0.05, FDR-corrected for multiple comparisons) of: a) frontal lobe (average thinning ranging from 8 to 11%), comprising the inferior part of the precentral sulcus, inferior, middle, and superior frontal sulci, and lateral and medial orbital sulci; b) limbic lobe (average thinning ranging from 8 to 12%), comprising the right cingulate sulcus (marginal branch), and anterior (ACC), middle-anterior (aMCC), pMCC, and posterior-ventral (vPCC) parts of the cingulate gyri and sulci; c) insula (thinning ranging from 9 to 13%), comprising the circular and lateral sulci; d) temporal lobe (average thinning ranging from 8 to 12%), comprising the superior temporal gyrus, and inferior and superior temporal sulci; e) parietal lobe (average thinning ranging from 9 to 11%), comprising the postcentral, intraparietal, transverse parietal and subparietal sulci; and f) occipital lobe (average thinning ranging from 9 to 10%), comprising the anterior occipital sulcus and preoccipital notch, lateral and medial occipito-temporal sulci, lingual sulcus, and parieto-occipital sulci. No regions with reduced cortical thickness were found in controls compared to ALS patients. The results did not change when the analysis was performed in limb-onset ALS patients compared with healthy controls (data not shown).

**Table 1:** Left hemisphere: regions with different mean cortical thickness in patients with amyotrophic lateral sclerosis compared with healthy controls.

Anatomical region	Healthy controls	ALS patients	% difference	p*
*Frontal lobe*				
Inferior part of the precentral sulcus	2.09±0.24	1.87±0.30	12	0.01
Inferior frontal sulcus	2.05±0.17	1.86±0.22	10	0.01
Middle frontal sulcus	2.15±0.19	1.94±0.20	10	0.003
Medial orbital sulcus	2.23±0.20	2.07±0.22	8	0.03
*Limbic lobe*				
Marginal branch of the cingulate sulcus	2.03±0.20	1.83±0.25	11	0.02
Middle-posterior part of the cingulate gyrus and sulcus (pMCC)	2.41±0.21	2.23±0.32	8	0.049
*Insula*				
Superior segment of the circular sulcus of the insula	2.50±0.20	2.30±0.32	8	0.04
Horizontal ramus of the anterior segment of the lateral sulcus (or fissure)	2.01±0.30	1.81±0.30	11.	0.049
*Temporal lobe*				
Anterior transverse collateral sulcus	2.89±0.31	2.60±0.46	11	0.049
*Parietal and occipital lobes*				
Postcentral sulcus	1.79±0.18	1.63±0.20	10	0.01
Intraparietal sulcus and transverse parietal sulci	1.90±0.16	1.74±0.22	9	0.02
Parieto-occipital sulcus	1.82±0.23	1.65±0.24	10	0.04

Values are mean thickness ± standard deviation (mm) or percent difference.

* p values refer to multivariate liner models, adjusting for subject's age (False-Discovery rate corrected).

**Table 2:** Right hemisphere: regions with different mean cortical thickness in patients with amyotrophic lateral sclerosis compared with healthy controls.

Anatomical region	Healthy controls	ALS patients	% difference	p*
*Frontal lobe*				
Inferior part of the precentral sulcus	2.08±0.20	1.87±0.31	11	0.01
Inferior frontal sulcus	2.02±0.18	1.85±0.20	9	0.003
Middle frontal sulcus	2.14±0.17	1.95±0.19	10	0.001
Superior frontal sulcus	2.29±0.23	2.10±0.31	9	0.03
Lateral orbital sulcus	2.15±0.27	1.98±0.25	9	0.03
Medial orbital sulcus	2.19±0.16	2.02±0.25	8	0.02
Orbital sulci	2.70±0.22	2.51±0.31	8	0.03
*Limbic lobe*				
Marginal branch of the cingulate sulcus	2.05±0.19	1.83±0.28	12	0.004
Anterior part of the cingulate gyrus and sulcus (ACC)	2.69±0.19	2.45±0.29	10	0.003
Middle-anterior part of the cingulate gyrus and sulcus (aMCC)	2.71±0.18	2.49±0.35	8	0.03
Middle-posterior part of the cingulate gyrus and sulcus (pMCC)	2.41±0.24	2.23±0.33	8	0.05
Posterior-ventral part of the cingulate gyrus (vPCC)	2.66±0.32	2.40±0.31	10	0.02
*Insula*				
Superior segment of the circular sulcus of the insula	2.60±0.20	2.34±0.32	11	0.003
Horizontal ramus of the anterior segment of the lateral sulcus (or fissure)	1.98±0.24	1.76±0.27	13	0.01
Posterior ramus of the lateral sulcus (or fissure)	2.11±0.21	1.93±0.30	9	0.03
*Temporal lobe*				
Planum temporal of the superior temporal gyrus	2.04±0.33	1.82±0.35	12	0.04
Inferior temporal sulcus	2.55±0.20	2.35±0.35	8	0.04
Superior temporal sulcus	2.28±0.14	2.11±0.29	8	0.03
*Parietal lobe*				
Postcentral sulcus	1.76±0.18	1.61±0.22	9	0.02
Intraparietal sulcus and transverse parietal sulci	1.89±0.16	1.70±0.18	11	0.001
Subparietal sulcus	2.34±0.23	2.13±0.34	10	0.03
*Occipital, occipito-temporal and occipito-parietal lobes*				
Anterior occipital sulcus and preoccipital notch	2.13±0.21	1.93±0.27	10	0.01
Lateral occipito-temporal sulcus	2.57±0.26	2.33±0.35	10	0.021
Medial occipito-temporal sulcus and lingual sulcus	2.23±0.19	2.05±0.28	9	0.02
Parieto-occipital sulci	1.86±0.19	1.69±0.24	9	0.02

Values are mean thickness ± standard deviation (mm) or percent difference.

* p values refer to multivariate liner models, adjusting for subject's age (False-Discovery rate corrected).

### Classification ability (Table 3)

**Table 3:** Sensorimotor, cognitive-frontal and cognitive-temporal mask mean cortical thickness in patients with amyotrophic lateral sclerosis compared with healthy controls.

Anatomical region	Healthy controls	ALS patients	% difference	p*	C-statistic
Left sensorimotor	2.14±0.16	1.96±0.22	9	0.0003	0.75
Right sensorimotor	2.18±0.13	1.98±0.24	10	0.0003	0.74
Left cognitive frontal	2.24±0.16	2.07±0.22	8	0.001	0.73
Right cognitive frontal	2.24±0.15	2.06±0.21	9	0.0003	0.74
Left cognitive temporal	2.25±0.16	2.11±0.29	7	0.03	0.65
Right cognitive temporal	2.31±0.14	2.13±0.29	8	0.003	0.68

Values are mean thickness ± standard deviation (mm) or percent difference.

* p values refer to multivariate liner models, adjusting for subject's age (False-Discovery rate corrected).

Abbreviations: ALS = amyotrophic lateral sclerosis; C index = Concordance index.

The mean cortical thickness of the left and right sensorimotor masks were able to classify correctly patients *vs.* controls with a C-statistic of 0.75 and 0.74, respectively (i.e., about 3/4 of subjects were classified correctly using an individual variable). The mean cortical thickness of the cognitive frontal masks had a C-statistic of 0.73 and 0.74 in the left and right hemispheres, respectively. The mean cortical thickness of the cognitive temporal mask classified correctly ALS *vs.* controls with a C-statistic of 0.65 and 0.68 in the left and right hemispheres, respectively.

### Relationship between cortical thickness and age

**Figure 3:**
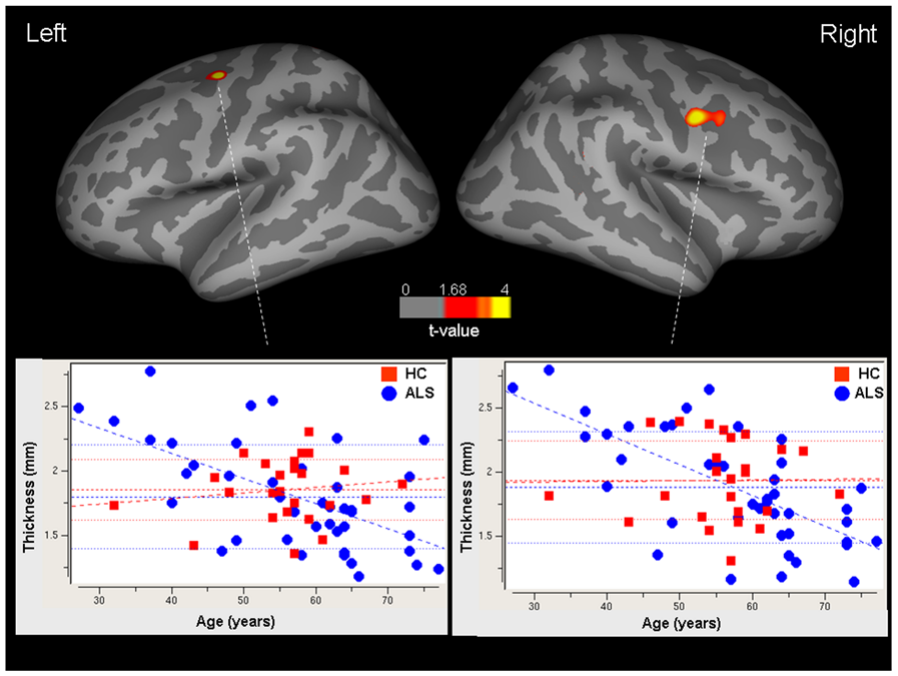
Relationship between cortical thickness and age. Top: vertex-by-vertex analysis; regions of significant age-by-group interaction with cortical thickness. Color bar represents t values (p<0.05). Bottom: scatterplots of the correlations between the cortical thickness measures and subject age in patients with amyotrophic lateral sclerosis (blue circles) and healthy controls (red squares).

The vertex-by-vertex analysis showed that there was a significant age-by-group interaction in the superior part of the left precentral sulcus (x −31, y −10, z 48; p = 0.05, uncorrected for multiple comparisons), and right precentral gyrus (x 48, y −49, z 9; p = 0.05, uncorrected for multiple comparisons) (Figure 3). The significant age-by-group interaction between cortical thickness measures and age was confirmed by the correlation analysis, which showed a stronger association in ALS patients relative to controls between age and mean cortical thickness of the left (heterogeneity p = 0.10; ALS r = −0.66, p<0.0001; controls r = −0.23, p = 0.26) and right (heterogeneity p = 0.09; ALS r = −0.61, p<0.0001; controls r = −0.24, p = 0.24) sensorimotor masks, and right cognitive temporal mask (heterogeneity p = 0.11; ALS r = −0.57, p<0.0001; controls r = −0.23, p = 0.26).

### Relationship between cortical thickness and disease progression rate

In ALS patients, previous disease progression rate correlated with the mean cortical thickness of the left sensorimotor mask (r = −0.33, p = 0.03).

## Discussion

Using a brain cortical thickness analysis in ALS patients, this study shows that these patients have a cortical thinning (of about 12%) of the precentral gyrus. The vertex-by-vertex analysis showed that in ALS patients cortical thinning was more prominent in the more cranial, dorsal portions of the primary motor cortex which contribute to control muscles of the trunk and extremities, in keeping with limb-onset clinical presentation in the majority of our cases. Previous *in vivo* MRI studies did not reach firm conclusions regarding the ability of imaging technology to detect primary motor cortical atrophy in ALS patients, since this was reported by some [6], [7], [8], [9], [16], [17], [18], but not all authors [10], [11]. There are several possible explanations for such a discrepancy. First, the intrinsic heterogeneity of the pathologic process should be considered [3], [20]. The histopathological abnormalities can be very subtle in some patients [3]. Furthermore, reactive gliosis in deep layers of the motor cortex has been described by most neuropathological studies [3], and it may occur at a degree enough to “mask” tissue loss. Second, the different clinical characteristics (i.e., physical disability, disease duration) of the cohorts of patients studied may be another possible explanation for the conflicting results. Finally, divergent results could be due to methodological factors related to the MRI analysis (i.e., cortical thickness measurement *vs.* VBM). Our findings agree with results of previous studies based on cortical thickness measurements [16], [17], [18]. By contrast, atrophy of the primary motor area has not been reported consistently by VBM studies [6], [7], [8], [9], [10], [11]. Although GM volume as measured by VBM is, by definition, the product of the surface area by thickness, it has been shown that differences in GM volume are driven almost exclusively by cortical surface rather than cortical thickness [21]. Since the size of cortical surface area is associated with the number of columns running perpendicularly to the surface of the brain [22], whereas cortical thickness is influenced by the number of cells within a column [23], it is likely that neurodegenerative processes may affect primarily thickness measurements rather than cortical surface size. In line with this hypothesis, cortical thickness provides more sensitive measures of age-associated GM decline compared with VBM in a large cohort of healthy individuals [24]. Moreover, cortical thickness was found to be superior to cortical surface area and volume in detecting the degenerative effects of ALS [18].

This is the first study showing that cortical thinning of the precentral gyrus is associated with the speed of disease progression: patients with faster rates of disease progression had experienced a more severe primary motor cortex decrease. Although there is an urgent need for markers with the potential to identify ALS patients with a more rapid rate of progression [25], optimal MRI measurements reflecting the speed of the clinical manifestations are still lacking. Previous studies using diffusion tensor MRI tractography reported a relationship between the structural connectivity properties of the corticospinal tracts and the rapidity of disease evolution in patients with ALS [26], [27]. Longitudinal cortical thickness studies are needed to validate the hypothesis that cortical thickness measurements and diffusion tensor MRI may be useful tools for monitoring disease progression in ALS, either natural or modified by treatment.

In addition to the cortical thinning of the precentral gyrus, cortical thickness measurements were found to be altered in multiple brain areas. Technical issues may explain the different results obtained using the vertex-by-vertex and the ROI approaches. The vertex-by-vertex analysis requires a stringent spatial overlapping of vertices showing a significant cortical thinning in order to capture a difference between groups, as it was the case for the precentral gyrus in our ALS patients. By contrast, ROI analysis derives the cortical thickness measurement of an individual cortical region by averaging values over all vertices within the given ROI, thus allowing to identify differences even in the case of subjects with a heterogeneous involvement of the cortical gyrus of interest. Our study suggests that ROI-based approach may be sensitive to detect the extramotor cortical involvement known to occur in ALS patients. When considering the extra-motor regions in the frontal, temporal, parietal and occipital lobes, ALS patients had up to 13% of cortical thinning relative to healthy controls. The evidence of a distributed involvement of extra-motor areas confirms several previous neuroimaging studies of ALS [5]. Early pathological studies have shown a significant reduction of the density of neurons in regions of the prefrontal cortex beyond the motor system, specifically the dorsolateral prefrontal cortex and the anterior cingulate cortex [28]. More recently, TAR DNA-binding protein pathology has been shown in multiple brain areas, including the neocortical and allocortical areas, nigrostriatal system, and cerebellum in ALS patients [29], [30]. While the involvement of the frontal, temporal and parietal lobes is in keeping with the known spectrum of cognitive and pathological overlap between ALS and FTD [31], none of our subjects had overt dementia. However, in the absence of a formal neuropsychological testing, we do not know whether such extra-motor cortical thinning was associated with some degree of cognitive impairment.

The “disease signature” approach to cortical morphometry [13], in which disease effects are mapped across the cortical mantle and then used to define ROI for hypothesis-driven analyses, is considered as a powerful methodological framework for studies of neurodegenerative diseases. Thinning of specific cortical areas known to be affected by Alzheimer's disease (AD) has been suggested as a way to identify individuals with mild cognitive impairment at a relatively high risk for imminent progression to AD [32]. In the present study, we wished to explore the potential of cortical abnormalities as diagnostic markers in patients with ALS. Not surprisingly, the variables that showed to be the strongest predictors of ALS *vs.* controls were those reflecting a thinning of the sensorimotor cortices, which were able to classify correctly about 3/4 of our subjects. However, a good diagnostic ability was also shown for the “cognitive” frontal and temporal areas, thus confirming that frontotemporal involvement is a consistent feature of ALS. It remains to be assessed the contribution of such measurements to a reliable identification of ALS individuals at a relatively high risk for subsequent development of cognitive impairment/dementia.

Our analysis also showed a significant association between age and cortical thickness measurements of sensorimotor and cognitive temporal regions in ALS patients. Aging is a major negative prognostic factor for ALS [33]. Several mechanisms have been proposed to explain the neuronal vulnerability to aging processes in ALS. Age-related increase in oxidative, metabolic, inflammatory activation or other type of homeostatic stress, resulting in the accumulation of damaged subcellular structures, DNA and proteins, are involved in disease mechanisms [34]. Our findings suggest that sensorimotor and temporal cortices of ALS patients may be particularly vulnerable to aging. Future studies are needed to clarify the relationship between aging and the disease-specific neurodegenerative processes in ALS patients.

## Materials and Methods

### Participants

Patients with sporadic ALS were recruited consecutively. To be included, patients had to have: a diagnosis of definite, probable, or probable laboratory-supported ALS according to the El Escorial revised criteria [35]; no family history of ALS; no clinical diagnosis of frontotemporal dementia (FTD) [36]; no any other major systemic, psychiatric or neurological illnesses; no other causes of focal or diffuse brain damage, including lacunae and other evidence of cerebrovascular disease at routine MRI scans; right-handedness [37]. Within 48 hours from MRI, functional status was assessed by the ALS Functional Rating Scale (ALSFRS-r) [38]. The rate of disease progression at the study entry was calculated as follows: (48 – ALSFRS-r score)/time from symptom onset [19]. All patients were on treatment with riluzole.

Forty-four ALS patients were included (20 women, mean age 58 years±12, mean disease duration 24 months ±17, Table 4). Thirty-six patients had a limb-onset, 7 patients had a bulbar-onset, and one patient had a bulbar+limb-onset disease. The mean ALSFRS-r score was 34±6 (range = 22–46). Mean disease progression rate was 0.94±0.91 (median = 0.58, range = 0.17–3.67). Twenty-six sex- and age-matched healthy subjects (13 women, mean age 56 years±8) served as controls (Table 4).

**Table 4:** Demographic and clinical data from healthy controls and patients with amyotrophic lateral sclerosis.

	Healthy controls	ALS patients	p*
N	26	44	-
Age [years]	56±8 (32–72)	58±12 (27–77)	0.26
Women, men	13, 13	20, 24	0.81
Limb/bulbar/limb+bulbar onset	-	36/7/1	-
Time from symptom onset [months]	-	24±17 (3–72)	-
ALSFRS-r	-	34±6 (22–46)	-
Disease progression rate° [units/months]	-	0.95±0.91 (0.17–3.67)	-

Numbers are mean ± standard deviation (range) or number. °Disease progression rate = (48-ALSFRS-r score)/time from symptom onset.

* Mann-Whitney-U-test or Fisher exact test as appropriate (see text fro further details). Abbreviations: ALS = amyotrophic lateral sclerosis; ALSFRS-r = ALS Functional Rating scale-revised.

### Ethics statement

The study was approved by the Ethics Committee of Scientific Institute and University Ospedale San Raffaele, Milan, Italy and a written informed consent was obtained from all subjects prior to study entry, according to the Declaration of Helsinki.

### MRI study

MRI scans were obtained on a 1.5 Tesla AVANTO system (Siemens, Erlangen, Germany), using four channel head coil. MRI sequences included: axial dual-echo (DE) turbo spin echo (TR/TEs = 3460/27-109, echo train length = 5, field of view [FOV] = 250 mm^2^, matrix size = 512×512; 35 contiguous, 4-mm thick slices); and sagittal 3D-T1-weighted magnetization prepared rapid acquisition gradient echo (MP-RAGE) (TR/TE = 2000/3.93, flip angle = 12°, FOV = 270 mm^2^, matrix size = 256×256, voxel size = 0.9×0.5×0.5 mm^3^, slab thickness = 187.2 mm).

MRI analysis was performed by an experienced observer, unaware of subjects' identity. Axial DE images were analyzed to assess the presence and location of areas with increased corticospinal tract signal intensity. Cortical reconstruction and thickness estimation was performed on the MP-RAGE images using the Freesurfer image analysis suite, version 4.5 (http://surfer.nmr.mgh.harvard.edu/) [12] on a 64-bit Linux CentOS 4. Briefly, after registration to the Talairach space and intensity normalization, the process involves an automatic skull stripping by using an hybrid method combining watershed algorithms and deformable surface models. Then, the white matter (WM)/GM boundary was tessellated and the surface was deformed following GM/cerebrospinal fluid (CSF) intensity gradients to optimally place WM/GM and GM/CSF borders. The results of these segmentations were inspected visually, and if needed, edited manually by adding control points. Finally, an automatic reconstruction of the cortex was produced and cortical thickness estimated by computing the average shortest distance between the WM boundary and the GM/CSF surface. Surface maps were generated following registration of all subjects' cortical reconstructions to a common average surface. Finally, surface maps were smoothed using a surface-based Gaussian kernel of 15 mm full width half-maximum. This smoothing kernel is slightly larger than the default kernel of Freesurfer (i.e., 10 mm) in order to ensure a better signal-to-noise ratio and a more precise alignment between input surfaces [39].

The Freesurfer processing stream generates thickness measures from 74 cortical and subcortical ROI per hemisphere, as described in Destrieux et al. [40].

### Statistical analysis

Statistical analysis was performed using SAS Release 9.1 (SAS Institute, Cary, NC, USA) and the Freesurfer image analysis suite. Between-group differences in demographic variables were assessed using the Fisher Exact Test or the Mann Whitney U test, for categorical and continuous variables, respectively. Normal distribution was checked using the Kolomogorv-Smirnov and Shapiro-Wilk tests and via graphical inspection of Q-Q plot.

Whole brain average cortical thickness was compared between groups using an analysis of covariance model, adjusting for subject's age. To investigate the regional differences of cortical thickness between ALS patients and controls, the following was performed: (*i*) a vertex-by-vertex analysis using a generalized linear model as implemented in Freesurfer. Cortical thickness was modeled as a function of group, controlling for age. Maps showing significant differences between ALS patients and controls and maps of age-by-group interaction were generated by thresholding the images of t statistics at a 0.05 significance level, uncorrected for multiple comparisons. (*ii*) The mean cortical thickness of the 74 ROI per hemisphere were compared between groups using multivariate linear models, adjusting for age. False-discovery rate (FDR) was controlled at a 0.05 significance level to correct for multiple comparisons. (*iii*) According to an *a priori* hypothesis based on available studies [5], and on the basis of the results of step (*ii*), three masks per hemisphere were derived, believed to constitute the cortical signature of ALS: the sensorimotor (inferior part of the precentral sulcus, middle-anterior and middle-posterior parts of the cingulate gyrus and sulcus, and postcentral sulcus), the “cognitive” frontal (inferior, middle and superior frontal sulci, and lateral and medial orbital sulci), and the “cognitive” temporal (planum temporal of the superior temporal gyrus, and inferior and superior temporal sulci). The mean cortical thickness of the three masks was obtained by averaging the thickness across the ROI part of a given mask, normalizing for ROI's area dimension. Cortical thicknesses of the three masks were compared between ALS patients and controls using multivariate linear models, adjusting for age (p<0.05, FDR-corrected). For the three masks per hemisphere, we also estimated the C-statistic (i.e., area under a receiver-operator characteristic curve) using a logistic regression analysis. The C-statistic quantifies the discriminatory ability of a considered variable in distinguishing groups of individuals.

In both controls and patients, correlations between the average cortical thickness measures of the sensorimotor, cognitive frontal and cognitive temporal masks and age were estimated using the Pearson coefficient. Adding an age-by-group interaction term into the multivariate linear models, the between-group heterogeneity of such correlations was assessed (p<0.10 was considered as suggestive of a different degree of association [41]).

In ALS patients, correlations between the mean cortical thickness measures of the sensorimotor, cognitive frontal and cognitive temporal masks and the disease progression rate were estimated using the Pearson coefficient, adjusting for age.
